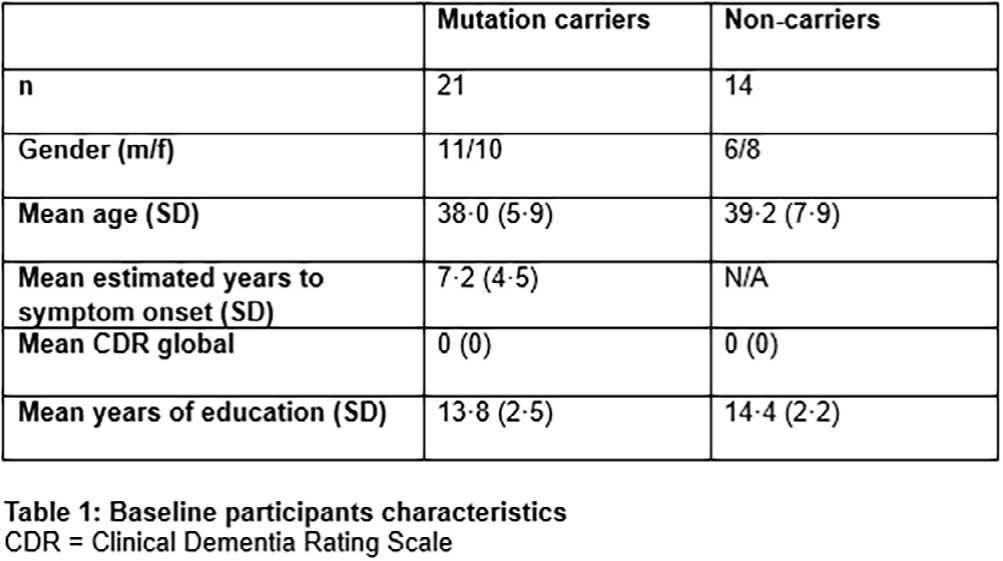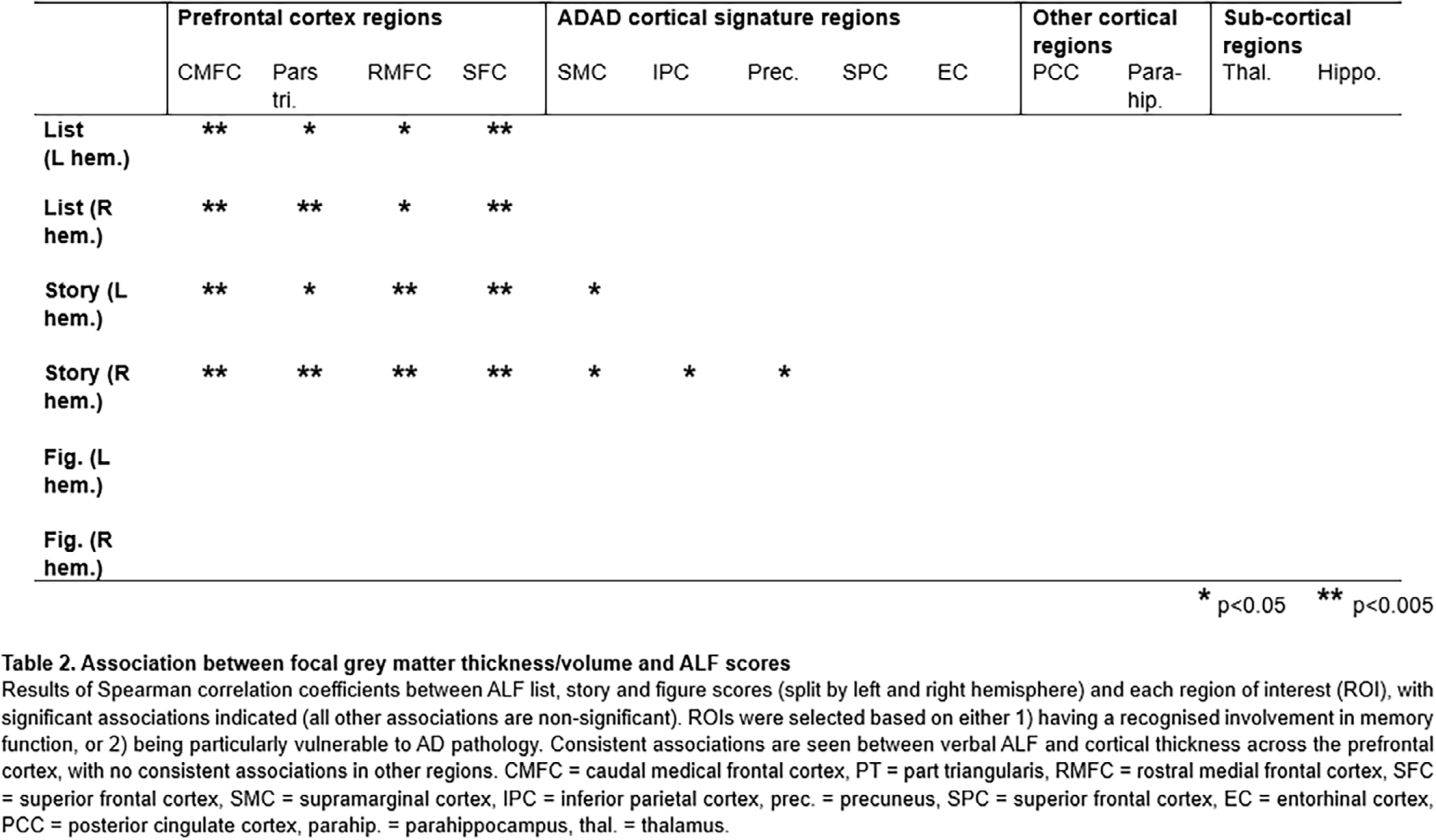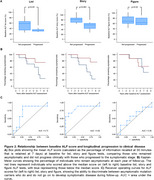# Neuroimaging correlates and prognostic utility of accelerated long‐term forgetting in presymptomatic autosomal dominant Alzheimer's disease

**DOI:** 10.1002/alz70857_105700

**Published:** 2025-12-25

**Authors:** Nicholas Magill, Christopher S Parker, Chloe Young, Rachana Tank, Damien Ferguson, Duncan Alston, Helen Rice, Antoinette O'Connor, Ian B. Malone, Kirsty Lu, Sebastian J Crutch, Nick C Fox, Philip SJ Weston

**Affiliations:** ^1^ Dementia Research Centre, Queen Square Institute of Neurology, London, United Kingdom; ^2^ London School of Hygeine & Tropical Medicine, London, United Kingdom; ^3^ UCL Hawkes Institute and Department of Computer Science, University College London, London, United Kingdom; ^4^ Dementia Research Centre, Queen Square Institute of Neurology, London, Greater London, United Kingdom; ^5^ Dementia Research Center UCL Institute of Neurology University College London, London, United Kingdom; ^6^ Dementia Research Centre, UCL Queen Square Institute of Neurology, London, London, United Kingdom; ^7^ Dementia Research Centre, UCL Queen Square Institute of Neurology, London, United Kingdom; ^8^ UK Dementia Research Institute, London, United Kingdom; ^9^ UK Dementia Research Institute at UCL, London, United Kingdom

## Abstract

**Background:**

In Alzheimer's disease (AD), sensitive measures of cognitive decline prior to overt symptoms are urgently needed. Accelerated long‐term forgetting (ALF), where information is retained normally over 10‐30 minutes but lost at an accelerated rate over subsequent days to weeks, has been identified cross‐sectionally in presymptomatic autosomal dominant and sporadic AD cohorts. However, two key questions remain: 1) what neuroanatomical changes underlie ALF; and 2) can ALF testing predict proximity to symptom onset?

**Method:**

Twenty‐one asymptomatic autosomal dominant AD (ADAD) mutation carriers and 14 non‐carriers (Table 1) underwent ALF assessment with 1) a list, 2) a story, and 3) a visual figure, with testing of 30‐minute and 7‐day recall. Baseline T_1_ and diffusion‐weighted MRI was obtained. Cortical thickness was estimated for 13 pre‐defined grey matter regions (Table 2), with streamline tractography used to assess associated structural connectivity. Participants underwent annual clinical follow‐up for a median of 7 years.

**Result:**

ALF test score was significantly lower in mutation carriers than non‐carriers across the three tests. In mutation carriers, verbal ALF (list and story) was associated with lower baseline cortical thickness in the prefrontal cortex (PFC) across four contiguous regions bilaterally (Table 2). This association was not present in non‐carriers. No associations were found between ALF and the thickness/volume of medial temporal lobe (MTL) structures. There was some association between ALF and PFC structural connectivity, although weaker than for cortical thickness. 9/20 mutation carriers developed symptoms during follow‐up. Those who became symptomatic had lower baseline ALF scores for both list (*p* = 0.03) and story (*p* <0.001) (Figure 1). Story ALF (AUC=0.82) and list ALF (AUC=0.73) discriminated between those who did and did not develop symptoms.

**Conclusion:**

Our results suggest that ALF in presymptomatic ADAD is related to early focal structural changes in the PFC, but not the MTL. Follow‐up demonstrated that ALF severity is not only associated with the presence of AD pathology but also is predictive of clinical onset, identifying those at highest risk of imminent decline. ALF testing is a biologically relevant measure that offers promise in aiding presymptomatic trial recruitment, as a presymptomatic cognitive endpoint, and potentially as a screening tool in the wider population.